# Bartter Syndrome Type 1 Presenting as Nephrogenic Diabetes Insipidus

**DOI:** 10.1155/2018/9175271

**Published:** 2018-02-21

**Authors:** Gianluca Vergine, Elena Fabbri, Annalisa Pedini, Silvana Tedeschi, Niccolò Borsa

**Affiliations:** ^1^Department of Pediatrics, Infermi Hospital, Rimini, Italy; ^2^Medical Genetics Laboratory, Ospedale Maggiore Policlinico, Mangiagalli e Regina Elena, Milan, Italy

## Abstract

Bartter syndrome (BS) type 1 (OMIM #601678) is a hereditary salt-losing renal tubular disorder characterized by hypokalemic metabolic alkalosis, hypercalciuria, nephrocalcinosis, polyuria, recurrent vomiting, and growth retardation. It is caused by loss-of-function mutations of the *SLC12A1* gene, encoding the furosemide-sensitive Na-K-Cl cotransporter. Recently, a phenotypic variability has been observed in patients with genetically determined BS, including absence of nephrocalcinosis, hypokalemia, and/or metabolic alkalosis in the first year of life as well as persistent metabolic acidosis mimicking distal renal tubular acidosis. We report the case of a child with a genetically determined diagnosis of Bartter syndrome type 1 who presented with a phenotype of nephrogenic diabetes insipidus, with severe hypernatremia and urinary concentrating defect. In these atypical cases, molecular analysis is mandatory to define the diagnosis, in order to establish the correct clinical and therapeutic management.

## 1. Introduction

Bartter syndrome is a hereditary salt-losing renal tubular disorder characterized by hypokalemic metabolic alkalosis, hypercalciuria, nephrocalcinosis, polyuria, and hyperreninemia with normal arterial blood pressure. It is due to several genetic defects affecting a number of ion transporters or channels along the ascending limb of the loop of Henle that play an important role in the blood volume regulation and in the reabsorption of NaCl. Depending on the affected transporters and channels, 5 types of Bartter syndrome (BS) can be identified. Type 1 BS (OMIM #601678) is caused by loss-of-function mutations of the *SLC12A1* gene, encoding the furosemide-sensitive Na-K-Cl cotransporter (NKCC2). BS types 1 and 2 account for most cases of the so-called severe antenatal or infantile Bartter syndrome, characterized by maternal polyhydramnios, prematurity, intrauterine and postnatal polyuria, recurrent vomiting, and growth retardation. Many mutations have been detected for each of the 5 genes, which may cause several different effects on protein synthesis [1–3]. The main hallmark of Bartter syndrome (BS) is hypokalemic metabolic alkalosis, but recently, a phenotypic variability has been observed in patients with genetically determined BS. Herein, we report the case of a patient with type 1 BS who initially presented with a phenotype of nephrogenic diabetes insipidus.

## 2. Case Presentation

The patient was a male infant born at 30 weeks of gestational age by cesarean section, after a pregnancy characterized by a marked maternal polyhydramnios. The birth weight was 1280 grams. The neonatal period was characterized by respiratory distress, hypovolemic acute renal failure, anemia, and retinopathy of prematurity. Renal function rapidly normalized after rehydratation. When discharged from the neonatal intensive care unit, the patient's renal function and plasma electrolytes were normal, except for a mild hypernatremia (maximum value 145 mEq/L). In the following months, laboratory investigations confirmed a more severe hypernatremia (150 to 160 mEq/L), whereas serum potassium level and acid-base balance were normal. The patient was referred to our centre at the age of one year, because of severe dehydration during an episode of fever. The child showed a severe growth delay with both weight and height below the 3rd percentile (weight 4.6 kg and height 60 cm), and at physical examination was evident a triangular facies with frontal bossing. The parents reported that, during the first year of life, the most important clinical findings were polyuria, intermittent high fever, recurrent vomiting, extreme irritability, and spasmodic need of water. Laboratory investigation showed a normal glomerular filtration rate (Schwartz formula calculated GFR 95 ml/min/1.73 m^2^) and severe hypernatremia (175 mEq/L), with normal serum potassium and blood gas. The tubular function test revealed a normal fractional excretion of sodium (FE Na 0.15%) and uric acid (FE uric acid 8%) and a normal tubular reabsorption of phosphate (TRP 92%), whereas a mild hypercalciuria was noted (calciuria/creatinuria 0.8, normal value for age 0.2). The transtubular potassium gradient was mildly increased (TTKG 11, normal value 5–10), suggesting a secondary hyperaldosteronism as a consequence of volume depletion. During the first days of hospitalization, we confirmed the presence of polyuria (2 L/day). In order to clarify the cause of polyuria, we carried out a fluid deprivation test, which showed a urinary concentration defect, and a vasopressin test, which confirmed the renal origin of the defect (urine osmolarity: 140→150 after vasopressin administration). At this point, given the clinical history and the laboratory test results, a diagnosis of nephrogenic diabetes insipidus (NDI) was hypothesized, although the presence of polyhydramnios and physical examination could be suggestive of Bartter syndrome. The child was started on hyperhydratation through a nasogastric tube, which determined a growth improvement and a normalization of serum sodium level (140 mEq/L). At the age of 18 months, for the first time, a mild low potassium blood level was noted (Table 1). Acid-base balance persisted normal, whereas an abdominal ultrasound showed a mild nephrocalcinosis and a gallbladder stone of 2.7 mm. These findings, together with the presence of polyhydramnios, premature delivery, and a characteristic facies, let us to suspect Bartter syndrome. We therefore performed genetic analysis of the *SLC12A1* and *KCNJ1* genes. Compound heterozygous mutations in the *SLC12A1* gene—c.730dupG (p.(Ala244Glyfs∗24)) and c.1432G>A (p.(Gly478Arg))—were found, confirming the diagnosis of Bartter syndrome type 1. Both the mutations are pathogenic: the first one truncates the protein synthesis and the second has been previously described by Vargas-Poussou et al. [4] and is highly conserved among the protein family from different species. No mutations were identified in the *KCNJ1* gene. The positive analysis of the *SLC12A1* gene made it unnecessary to test *AVPR2* or *AQP2* genes.

## 3. Discussion

Recently, phenotypic variability in patients with Bartter syndrome has been reported. Pressler et al. [5] described a mild and late-onset Bartter syndrome type 1 in two brothers of 13 and 15 years of age. Molecular genetic analyses revealed that the brothers were compound heterozygotes for mutations in the *SLC12A1* gene. Because one of the mutations only partially impaired NKCC2 function, variable degrees of NKCC2 dysfunction may explain a mild and late-onset phenotype. Moreover, some reports of transient neonatal hyperkalemia and metabolic acidosis in patients with antenatal Bartter syndrome type 2 are described, suggesting an erroneous diagnosis of pseudohypoaldosteronism type 1 [6]. Bettinelli et al. [7] reported a series of eight patients with atypical genetically determined Bartter syndrome type 1, including absence of nephrocalcinosis, absence of hypokalemia, and/or metabolic alkalosis in the first year of life as well as persistent metabolic acidosis mimicking distal renal tubular acidosis. They also described a patient with BS type 1 that presented hypernatremia, hyperchloremia, and a severe urinary concentration defect mimicking NDI, as in our case report. Although a urinary concentration defect is usually present in Bartter syndrome, persistent and severe hypernatremia is a very unusual finding. To our knowledge, our case is the third Bartter syndrome patient presenting with a clinical picture of nephrogenic diabetes insipidus without hypokalemia [7, 8]. Primary inherited NDI is due to mutations in either *AVPR2* or *AQP2*. NDI can also occur as a secondary complication, most commonly from obstructive uropathy or chronic lithium therapy. Recently, Bockenhauer et al. [8, 9] described the occurrence of NDI in four patients with inherited tubulopathy, including one case in a child with Bartter syndrome. Molecular analysis of the *AVPR2* and *AQP2* genes was unremarkable in all patients, suggesting that NDI was a secondary complication rather than an incidentally second inherited disease. In all cases, administration of vasopressin failed to increase urine osmolarity. Bartter syndrome is perhaps the most surprising diagnosis to underlie secondary NDI. The mechanism is not understood but probably other factors are implicated, including hypokalemia, hypercalciuria, nephrocalcinosis, and high urinary flow and pressure that may cause changes in the expression of *AQP2* [10, 11]. In fact, hypokalemia is typically associated with decreased *AQP2* expression, hypercalciuria affects renal concentrating ability via activation of the calcium-sensing receptor, and nephrocalcinosis may impair water reabsorption.

The sister of the patient with Bartter syndrome reported by Bockenhauer also suffers from Bartter syndrome with the same mutation identified, but she did not develop a secondary NDI despite identical *KCNJ1* genotype. The authors stated that there is no correlation between mutation type and secondary NDI, assuming a possible role of environmental factors, or different genetic backgrounds (additional variants). Furthermore, individual phenotypic variability is frequent in Bartter and Gitelman patients and can be explained by gender, personal compensatory mechanisms, or dietary habits. Nevertheless, we cannot fail to note that Bartter syndrome type 1 patient described by Bettinelli and most of the cases described by Bockenhauer carry severe frameshift mutations; likewise, our patient shows a heterozygous nucleotide duplication resulting in a premature truncated protein in the third NKCC2 transmembrane domain.

Recently, Brugnara et al. also reported a patient with a genetic combination of Gitelman syndrome and autosomal dominant diabetes insipidus [12]. Regardless of the mechanism, the diagnosis of NDI as a secondary complication is important, as these patients are at risk of hypernatremic dehydration. Early recognition and appropriate management are thus critical. In fact, Bartter syndrome is a salt-wasting disorder and requires supplementation with large amount of salt, whereas in NDI, salt restriction is crucial to minimize renal solute load and thus urine output. Moreover, NDI requires treatment with thiazide diuretics that can be very dangerous in patients with Bartter syndrome.

## 4. Conclusion

In conclusion, hypokalemia and metabolic alkalosis may not be found during the first year of life of Bartter syndrome patients. Furthermore, as in the present case, type 1 Bartter syndrome may be characterized by severe hypernatremia and urinary concentration defect, suggesting an erroneous diagnosis of nephrogenic diabetes insipidus. In these atypical cases, molecular analysis is necessary to define the diagnosis.

## Figures and Tables

**Table 1:** Laboratory investigation at onset and during follow-up.

Age (months)	12	13	15	18	24
		(DDAVP)			
Plasma					
Na (mEq/L)	175	150	140	142	138
K (mEq/L)	4	3.9	3.8	3.3	3.3
HCO_3_	24	25	26	27	27
BE	−1	−1	0	−1	2
Urine					
Ca_ur_/Creat_ur_	0.8	0.6	0.4	0.7	0.5
Osm_ur_ (mOsmol/kg)		140 (pre) 150 (post)			

## Abbreviations

BS: Bartter syndrome
FE: Fractional excretion
TTKG: Transtubular potassium gradient
NDI: Nephrogenic diabetes insipidus.
